# Refusal of Benefits

**Published:** 1913-11-29

**Authors:** Sidney Webb

**Affiliations:** 37 Norfolk Street, London, W.C.


### THE EDITOR'S LETTER-BOX.

### The Refusal of Benefits.

*To the Editor of* The Hospital.

Sir,—The Committee of Inquiry into the Working of the Insurance Act, instituted by the Fabian Research Department, has reason to believe that some insured persons are being refused the benefits to which the Act entitles them, and that many poor people feel unable to resist what seems to them oppression. Will you allow me, through your columns, to invite all who know of any case of what seems to be wrongful refusal of benefits to write (in strict confidence) to me, giving full particulars?

Information is wanted as to the following cases, among others :—

1. Refusal of sickness benefit when the proved incapacity to work is caused by pregnancy, on the ground that this is not a disease. But the Act entitles to benefit for every "disablement," without specific disease; and Section 110 of the "Commissioners' Handbook" explicitly recognises incapacity caused by the disablement of pregnancy as a valid ground for sickness benefit.

2. Restriction of sickness benefit in pregnancy and maternity to a maximum of four weeks. There is no limitation of sickness benefit in the Act for members of approved societies, apart from the twenty-six weeks' maximum.

3. Refusal to continue sickness benefit when the doctor certifies incapacity to work, on the ground that the patient is found (1) breaking the rules for persons on benefit (thereby incurring a fine only); (2) doing light household work deemed by the doctor not harmful; (3) even "looking after her children."

4. Refusal of sickness benefit on the ground that, although unable to follow his own occupation, the patient is capable of "some" work. This amounts to a parody of the law, for no one is ever incapable of *any* work whatsoever, unless he is continuously unconscious. (Even the bedridden paralytic may knit, or pick peas, or write a book!)

5. Failure to provide the "adequate medical attendance and treatment" guaranteed by the Act for all cases without any exception. Particulars are desired of all cases in which (1) panel doctors have required payment for services beyond the scope of their contract with the Insurance Committee; (2) panel doctors have been unable to give the treatment required—*e.g.*, surgery, eye cases, etc.; (3) hospital treatment was required and not obtained; (4) the prescribed drugs or appliances have not been supplied free of charge; and (5) appliances not included in the Commissioners' list (trusses, elastic bandages, spectacles, crutches, etc.) have been required and refused.

6. Failure to provide what is required by tuberculosis patients, such as (1) inability to gain admission to sanatorium; (2) refusal of the necessary means of adequate domiciliary treatment, such as open-air sleeping arrangements or necessary ventilation; (3) refusal to allow nourishing food, such as milk and eggs, which the Act empowers the panel doctor to prescribe under certain circumstances.

7. Refusal of maternity benefit or of sickness benefit in connection therewith, on the ground of "misconduct" or because of alleged breach of rules.

8. Expulsion from approved society, for instance, on ground of "withholding material information."

The Committee welcomes testimony on both sides, and would like equally illustrative cases in which, under the circumstances indicated above, benefits had been given or continued and instances of their advantageous results.—I am, etc., Sidney Webb.

37 Norfolk Street, London, W.C.

